# A novel porcine dentin-derived bone graft material provides effective site stability for implant placement after tooth extraction: a randomized controlled clinical trial

**DOI:** 10.1007/s00784-023-04888-5

**Published:** 2023-02-24

**Authors:** Lari Sapoznikov, Doron Haim, Barbara Zavan, Gérard Scortecci, Martin F. Humphrey

**Affiliations:** 1 Private Practice, Tel Aviv, Israel; 2 Shamir Medical Center, 70300 Zerifin, Israel; 3 Department of Translational Medicine, University of Ferrara, 44121 Ferrara, Italy; 4 Basal Implantology Program, Department of Maxillo-Facial Surgery, School of Medicine, University Côte d’Azur, 06000 Nice, France; 5 Private Consultant, Keferstrasse 33, 80802 Munich, Germany

**Keywords:** Bone substitutes, Alveolar bone loss, Dentin, Dental implants, Clinical trial

## Abstract

**Objectives:**

Assessment of the clinical performance of a porcine dentin-derived particulate bone graft material for bone regeneration after tooth extraction with implant placement at 4 months, in comparison to a commercially available porcine bone-derived graft.

**Material and methods:**

This study was a randomized, parallel-group, semi-double-blinded clinical trial evaluating the clinical safety, tolerability, and performance of Ivory Dentin Graft™ in comparison with a commercial bone-derived material in alveolar ridge preservation following tooth extraction (registered at ClinicalTrials.gov, May 12th, 2017, Identifier NCT03150472). Extraction sites were grafted with test or comparator material and a titanium implant placed at 4 months after taking a graft site biopsy. Primary endpoints were the extent of new bone growth and bone-graft integration at 4 months.

**Results:**

The dentin graft material had statistically significantly more new bone formation (60.75% vs 42.81%, *p* = 0.0084, *N* = 20 vs 16), better bone-graft integration scores (good integration in 85% vs 40%, *p* = 0.0066), and higher mean radiodensity of the bone (981.5HU vs 727.7HU, *p* = 0.0011) at the graft site compared to the bone-derived material. The mean implant insertion torque force was similar for the dentin and bone materials (34.75 Ncm vs 34.06 Ncm). Titanium implant placement was successful in 95% of patients with the dentin graft material compared to 81.25% for the bone graft. Both materials had similar clinical safety and tolerability as determined by adverse events and local site reactions. Physician-assessed ease of grafting and ease of implant placement on a 10-point scale showed no statistical differences (8.78 vs 8.27, *p* = 0.2355; 8.05 vs 8.75, *p* = 0.1118, respectively).

**Conclusions:**

A porcine dentin-derived bone graft material has clinical safety, tolerability, and performance for implant placement at 4 months after tooth extraction at least as good as a commercial bone-derived material.

**Clinical relevance:**

The availability of porcine dentin-derived bone graft material allows wider use of dentin-derived material which has so far only been available in the form of autologous dentin from the patient’s own teeth.

## Introduction

The use of osseointegrated dental implants has become a standard for the prosthetic rehabilitation of fully and partially edentulous patients, providing excellent long-term functional and esthetic outcomes [1–5]. Where necessary, to optimize the repair of dental bone defects and to adequately support implants, a wide variety of bone graft materials have been developed and are available on the market [6]. The gold standard material is autologous bone as it contains the patient’s own cells, growth factors, and biomolecules needed for osteogenesis and has the highest degree of biological safety, biocompatibility, and matching mechanical properties [6–8]. In practice, however, a second surgery is generally required to harvest autologous bone, which is often not acceptable in the context of dental procedures. The bone must also be adequately prepared, thus increasing procedural complexity, and material resorption is often variable and thus does not always match the requirements for optimal repair. To provide practical alternatives to autologous bone, a range of materials have been developed including allogenic bone, xenogeneic bone, synthetic bio-ceramics, and synthetic polymers or composite biomaterials. None of these materials fulfills all the desired requirements but has different strengths and weaknesses [8]. Recently, there has been considerable interest in the use of tooth dentin-derived material due to the unique properties of dentin and clinical evidence from the use of autologous dentin from the patient’s own teeth showing that it is an efficacious bone graft material [9–17]. Dentin is harder than bone and has a regular porous structure due to the tubules and has the ability to form intimate contact with host bone in the process of ankylosis resulting in structurally stable contacts that are only very slowly resorbed by external replacement resorption in which the resorbed dentin is replaced by bone in a natural turnover process without inflammation [18–20]. The clinical experience with autologous dentin has shown that it is an effective bone graft material for dental use, but, in many circumstances, there is insufficient material. We have therefore developed a porcine tooth-derived dentin material with retained organic matrix using stringent quality controls to ensure safety and biocompatibility. A problem for determining the relative effectiveness of bone graft materials is generally the lack of comparative randomized, controlled clinical trials to support their use and assist the clinician in choosing the appropriate product [21]. We have therefore performed a clinical trial to compare our novel porcine dentin-derived material with a clinically established porcine bone-derived material.

This publication describes the results of a prospective, randomized, semi-blinded comparative trial of a novel bone substitute material, Ivory Dentin Graft™ (referred to as “Dentin”) in terms of the primary outcomes of the amount of new bone formation (percent area of woven bone) and the degree of direct contact between the graft and bone (qualitative histological assessment) at the graft site at 4 months after grafting. A number of other clinically relevant outcomes were also assessed as secondary endpoints. Ivory Dentin Graft™ is a novel xenogeneic origin, osteoconductive, osteoinductive, and slowly bioresorbable bone graft material for the repair or augmentation of bone defects in dental procedures (Table 1). The material is derived from porcine teeth obtained from health-controlled animals with a strictly controlled process that eliminates potential infectious agents, and it consists of sterile porous particles or granules of hydroxyapatite, which retain the natural form of the porcine dentin including the regularly spaced dentin tubules and also retains the natural protein matrix which largely consists of collagen but also potentially contains growth factors important for regeneration [17, 22, 23]. Ivory Dentin Graft™ has been shown to be effective and biocompatible in both standard and clinically relevant animal models of bone grafting and is prepared using a controlled process ensuring adequate quality and safety for human use (Table 1, unpublished data). Ivory Dentin Graft™ was compared with Gen-Os® (referred to as bone) because Gen-Os® is a bone graft material which has been on the European market for many years, has a large number of publications documenting its clinical efficacy and safety in a variety of indications, and is also a porcine-derived material with some retained collagen, but is derived from bone. The graft materials were examined in patients requiring bone grafting after molar or premolar extraction prior to implant placement.

**Table 1:** Properties and specifications of Ivory Dentin Graft™

Parameter		Property/specification
Raw material	Source	Porcine teeth
	Composition	Granules of hydroxyapatite, which retain the natural form of porcine dentin as well as the natural protein matrix, which largely consists of porcine collagen
Material properties	Physical form	80% tubule diameters 0.7–1.5 µm 20% coarsely porous pore size 2–15 µm
	Particle size	300–900 µm
	Vickers hardness	73 HVO.3 ± 14 HVO.3
	Ca:PO_4_	1.59–1.67
	Micro-structure	Scanning electron microscope shows porous structure consistent with original dentin
	Organic content	89% type I collagen, 1% proteoglycans, 10% others (phospho-, GLA-, glycoprotein, osteonectin, osteopontin, dentin sialoprotein), partially degraded
Implant properties	Resorption	The dentin material is expected to be slowly resorbed (5–7 years)
	Bone growth	An in vivo comparative study in a clinically relevant porcine model involving grafting into extraction sockets and sub-periosteum pouches showed performance similar to OsteoBiol Gen-Os®. At 10 weeks, the grafted areas were solid, dense, and stable with no sign of loose particles. Homogeneous radio-opacity was observed by X-ray in the grafted sites. Histologic analysis showed new bone growth in close apposition to the dentin particles
	Biocompatibility	In the porcine extraction socket and sub-periosteum pouch model, tight apposition of new bone growth with the dentin particles with only moderate inflammation consistent with healing processes was observed. Particles were more slowly resorbed than Gen-Os® In a rabbit femoral condyle defect combined implantation and systemic toxicity study comparing with the commercial material Gen-Os®, no signs of intrinsically adverse local reactions, no local draining lymph node reaction, and no signs of systemic toxicity were found at both weeks 4 and 12 after implantation There was no evidence of in vitro cytotoxicity of extracts (medium incubation for 72 h at 37 °C) in a standard mouse fibroblast L929 model using cellular morphology and the MTT assay as endpoints All materials are of natural biological origin that have not been modified in a substantial way and are therefore expected to be resorbed/degraded similar to natural tissue components without concerns for toxicity, including genotoxicity or reproductive toxicity Therefore, the material is considered to be biocompatible
Usage		Single use, do not re-sterilize
Use period		Permanent (implanted)
Shelf life		5 years from sterilization date
Storage conditions		Store protected from direct sunlight or contact with hot surfaces in a dry environment at temperatures between + 5 and + 30 °C
Sterility		Gamma irradiation, bioburden: max. 220 colony-forming units/device, max. 20 endotoxin units/device

The study was powered to demonstrate the non-inferiority of Ivory Dentin Graft™ to the comparator in terms of the amount of new bone formation and the integration of the graft material with the host bone in the core biopsy taken just prior to implant placement at 4 months. It is intended to follow up on the implant success over the longer term, but this publication focuses on the events up to and including implant placement as this includes the most critical events for assessing bone graft material properties.

## Material and methods

### Study design and treatment

The study is a randomized, parallel-group, semi-double-blinded clinical trial to evaluate the safety and effectiveness of Ivory Dentin Graft™ (“Dentin,” 1.0 g in either vials or syringes) in comparison with the active comparator OsteoBiol Gen-Os® (Bone, 1.0-g vials) in adult subjects requiring alveolar ridge preservation following mandibular pre-molar or molar tooth extraction. A comparison with a commercially available active comparator was done because this study was performed to support marketing authorization which requires a demonstration of non-inferiority to existing treatments and provides a higher hurdle than a negative control group comparison. An additional negative control group was considered beyond the scope of the study particularly due to ethical considerations and problems with ensuring a comparable patient group. The study was sponsored by Ivory Graft Ltd., Tel Aviv, Israel, and performed at a single clinical center, the Assaf Harofeh Medical Center, Rishon Lezion, Israel, under the supervision of the principal investigator Dr. Doron Haim. The study conforms to the CONSORT and Cochrane guidelines.

The protocol was in accordance with the Helsinki Declaration and the Fortaleza revisions, ICH E6 (R2), and ISO 14155:2011 and was approved by the Institutional Review Board for Clinical Research at Asaf Harofeh Medical Center (Approval No. 0102–17-ASF) and the Israeli Ministry of Health (Approval No. 20173907). Written informed consents were obtained from all participants after provision of a detailed explanation of the protocol and the benefits or risks of participation. The study was registered at ClinicalTrials.gov on May 12th, 2017 (Identifier NCT03150472), prior to the enrolment of the first subject.

This study was semi-double-blinded. Only the study staff member performing the grafting procedure, the study coordinator, and the study CRO were unblinded to the type of graft applied. The study assessors were located externally from the medical center, independent, and kept blinded to the subject allocations.

**Fig. 1:**
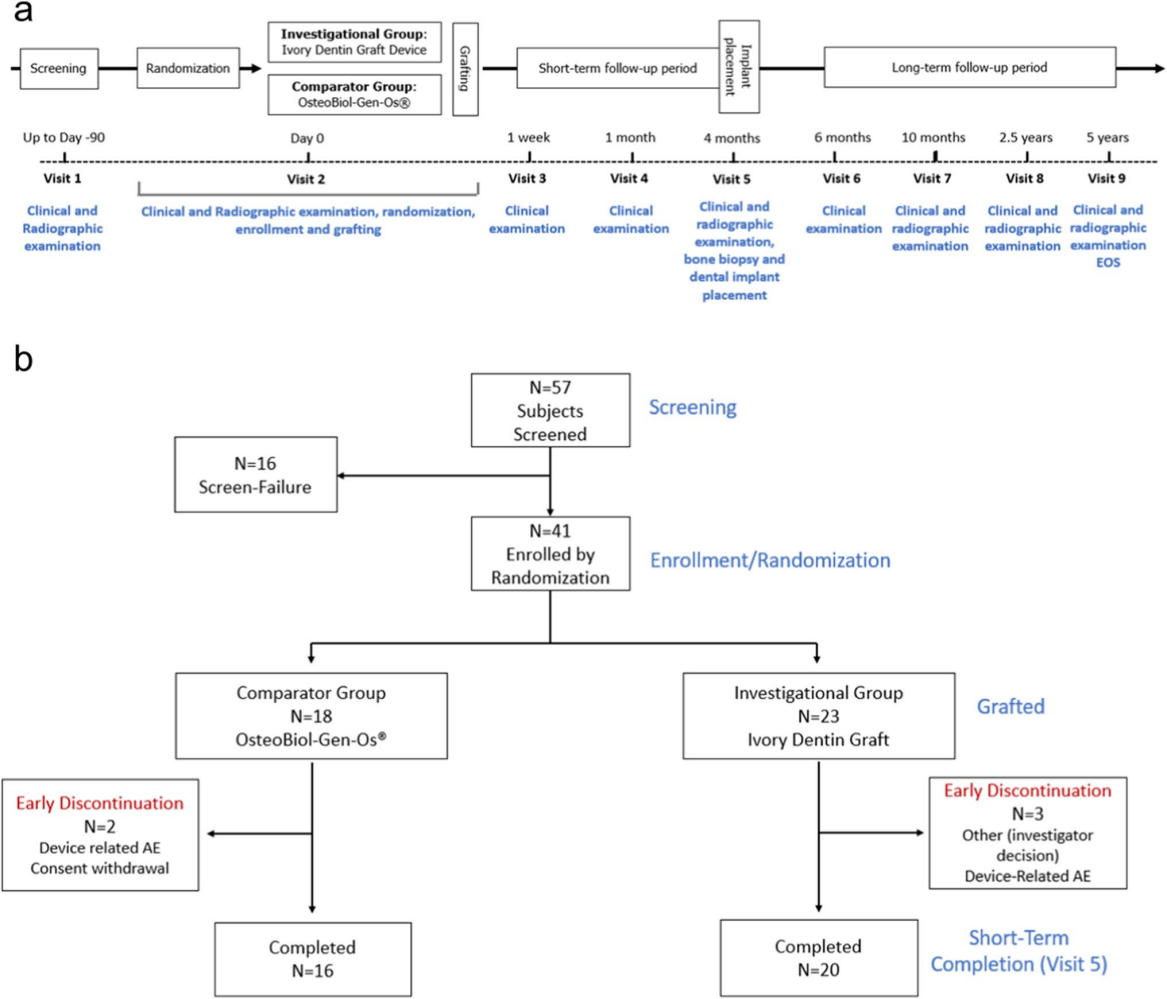
**a** Study schema and **b** subject disposition. This publication reports the findings up to the time of implant placement at 4 months (visit 5). Thirty-six subjects completed the study up to visit 5

Grafting procedures were conducted following mandibular pre-molar or molar tooth extraction. The sockets were required to have 4 walls with an alveolar ridge height of not less than 10 mm from the gingival margin to the mandibular nerve canal and a width of not less than 5 mm from buccal to lingual cortical plates. After ensuring compliance with the entry criteria, eligible patients were planned to be randomly assigned by the study site at a ratio of 1:1 to dentin or bone graft groups using Castor’s electronic data capture (EDC) web software and baseline examinations performed (Fig. 1a). Due to an error of entering screen failures into the system, the allocation deviated from 1:1 initially, but this was corrected by protocol amendment to a 4:1 ratio resulting in an overall equal allocation to the groups. The dentin and bone graft materials were prepared according to the appropriate information for use for each subject. The granule mix was applied to the prepared socket and covered with the same type of collagen membrane (Janson® fleece) to hold the graft material in place. Routine clinical examinations were performed at 1 week and 1 month after grafting (Fig. 1a).

A dental implant was placed at the end of the short-term follow-up period—4 months after bone graft (Fig. 1a). In cases where the graft site was found to be inadequate, re-grafting of the same graft material (dentin or bone) was performed, and the same follow-up schedule was employed. The 4-month follow-up period was chosen because according to consensus reviews, 3–4 months is the minimal healing period for alveolar ridge preservation procedures with up to 6 months required depending on the material- and patient-specific factors. For the dentin-derived material, the retained collagen and process of ankylosis-like contacts between the host bone and the material results in a stable graft site relatively early. The bone material with retained collagen also results in relatively rapid host bone ingrowth, and a number of published studies had evaluated the graft site histology at 3–4 months after grafting. Also, the graft sites were in the mandible which supports earlier graft site repair due to the higher proportion of compact bone and more rapid vascularization.

### Population

The patient selection criteria were standard for this type of study (Table 2). The criteria were selected to ensure clinical relevance and sensitivity for endpoint measurements and to exclude pre-existing conditions that would be contraindications for such procedures.

**Table 2:** Clinical study methods. Patient selection criteria and study endpoints

Inclusion criteria	1. Male or female patient 18 up to 80 years 2. Patient requiring at least one implant placement following mandibular pre-molar or molar tooth extraction 3. Alveolar mandibular ridge (empty socket): - Height: not less than 10 mm, from the gingival margin to the mandibular nerve canal—as seen in the screening CT scan - Width: not less than 5 mm, from buccal to lingual cortical plates—as seen in the screening CT scan 4. Ability to give informed consent for the study by a patient or legal guardian 5. Willingness to undergo 7 follow-up visits: 1 week; 1, 4, 6, and 10 months; 2.5 years; and 5 years following dental graft implantation, as well as unscheduled sick visits
Exclusion criteria	1. Pregnancy (all women of childbearing age would be questioned and told by the consenting physician regarding that criteria) 2. Known or suspected hypersensitivity to the constituents of the bone graft material (for example, porcine collagen) 3. Pathologies or conditions contraindicating surgery or presenting with active acute or chronic infections excluding periapical granuloma (for example, osteomyelitis, sinusitis), uncontrolled diabetes 4. Immunologic disorders or auto-immune pathologies, in particular, elderly subjects 5. Serious bone diseases of endocrine etiology 6. Serious disturbances of bone metabolism 7. Ongoing treatment with gluco- or mineralo-corticoids or with agents affecting calcium metabolism (e.g., calcitonin, bisphosphonates) 8. Irradiation therapy, chemotherapy, or immunosuppressive therapy in the last 5 years 9. Malignancies 10. Severe parafunction (bruxism and clenching) 11. Poor oral hygiene or active periodontitis 12. Heavy tobacco smoking habit (> 10 cigarettes per day)
Primary efficacy endpoints	1. Amount of new bone formation (mean area of mineralized and non-mineralized tissue) in alveolar bone core biopsies—“woven bone” (ratio 0–100%) at 4 months after grafting 2. Bone–graft material integration to host bone score in alveolar bone core biopsies at 4 months after grafting: - 1 — Poor: no signs of new bone-to-graft interface visible - 2 — Intermediate: minimal and focal signs of new bone-to-graft interface visible - 3 — Good: abundant new bone-to-graft interface visible
Secondary efficacy endpoints	1. Alveolar bone strength (torque measurement) at 4 months after grafting 2. Alveolar bone radiodensity (Hounsfield scale) calculated by volumetric CT imaging at 4 months after bone grafting 3. Success of dental implant placement in a rigid post-bone grafting site, defined by immediate dental implant stability after 4 months from bone grafting (visit 5) 4. Changes from baseline in alveolar bone height (depth reduction) at 4 months, measured at mesial and distal root surface [in millimeters] on Posterior to Anterior (PA) radiographs or by CT 5. Changes from baseline in alveolar bone width (horizontal bone gain or loss) (in millimeters) at 4 months on posterior to anterior (PA) radiographs or by CT
Safety endpoints	1. Number of participants with treatment-related adverse events as assessed by Common Terminology Criteria for Adverse Events (CTCAE) v4.0 through study completion (short-term and long-term) 2. Number of participants requiring unscheduled hospital visit related to the study procedure through study completion (short-term and long-term) 3. Safety and tolerability following grafting (graft site infection, insufficient healing of graft site, excessive bleeding, and wound dehiscence) over both short-term and long-term
Usability endpoints	1. Physician assessment of the ease of graft placement using a 10-point satisfaction scale 2. Physician assessment of the ease of implant placement using a 10-point satisfaction scale

### Study endpoints

The primary outcomes of the study were the amount of new bone formation (percent area of woven bone) and the degree of direct contact between the graft and bone (qualitative histological assessment) at the graft site at 4 months after grafting. All study endpoints are summarized in Table 2. The primary efficacy endpoints [24–26] and also measures of alveolar ridge dimensions [24, 25, 27, 28] have been validated by historical studies on a variety of bone substitutes (synthetic, autograft, allograft, xenografts). The main primary endpoint, which was used for the statistical power calculation, was the percent area of new bone formation (“woven bone”) in sections of 4–5 mm trephine bur biopsies taken through the center of the graft site during dental implant placement at 4 months (± 21 days) post-grafting. This quantifies the degree of host bone ingrowth into the graft site. The second primary endpoint was an assessment of the degree of direct contact between the host bone and graft material in the biopsy sections using a 3-point qualitative scale (see Table 2). This is a measure of the bone–graft integration which is important for graft site stability [29, 30]. Histological assessments were performed by L.E.M Laboratory Ltd., Nes-Tziyona, Israel.

Bone graft site quality was also assessed by the insertion torque during implant placement. Dental implant insertion torque has been shown to be correlated with bone density and is one of the most important factors for successful implant placement in a post-grafting site [31, 32]. Bone density was evaluated using a volumetric CBCT imaging dental software (OnDemand3D™), which measured the radiodensity of the alveolar bone core using CBCT images obtained prior to dental implant placement. This method is objective, reliable, and offers the best radiographic method for the morphological and qualitative analysis of the residual bone [31, 33–37].

A further efficacy endpoint was the success of the dental implant placement procedure. A successful implant placement event was defined as an event not requiring the addition of graft material during implant placement or postponement of the implant placement due to re-grafting.

Efficacy was also examined by measuring the changes in alveolar ridge dimensions at the graft site. Alveolar bone height at mesial and distal root surface and alveolar bone width was measured on posterior to anterior (PA) radiographs by cone beam computer tomography (CBCT scan) prior to bone grafting and just prior to implant placement at 4 months to accurately measure the changes of the alveolar ridge dimensions during the short-term period. CBCT is considered the best method to evaluate the changes in the alveolar ridges and ridge morphology, following tooth extraction and grafting, as well as for pre-implant surgical design and implant diameter selection. CBCT is considered more accurate than computer tomography (CT) and safer, as the patients are exposed to lower levels of radiation.

The ease of the graft placement procedure and the implant placement (scored by the physician performing the procedure using a 10-point satisfaction scale, 10 = easiest, 0 = complicated) was also recorded.

Safety parameters were also systematically collected including adverse events (AEs), unscheduled visits, and local tolerability (Table 2). All AEs were coded using the Medical Dictionary for Regulatory Activities (MedDRA) terminology.

### Sample size consideration

For calculation of the sample size, the null hypothesis was that Ivory Dentin Graft™ and Gen-Os® are not equivalent (Gen-Os® is better than Ivory Dentin Graft™), and the alternative hypothesis was that Ivory Dentin Graft is not inferior to Gen-Os® in respect of the study endpoint new bone formation. The statistical calculation based on demonstrating non-inferiority assumed a difference of up to 30% in the area of woven bone between the treatments to be equivalent with a standard deviation of 32%.

In a sample of 15 subjects per group, a difference of up to 30% in the mean woven bone between the treatment groups is considered equivalent with a 5% significance level and 80% statistical power.

Assuming a dropout rate of ~ 30% required 41 subjects to be recruited and grafted with either the dentin graft or the active comparator bone graft, to ensure a final sample size of 30 study completers (15 per group).

## Results

The first subject was enrolled on December 12th, 2017, and the last subject completed visit 5 on January 14th, 2020. Out of 57 subjects screened, a total of 41 subjects were randomized to the treatment groups (Fig. 1b). The two groups had similar age ranges (dentin: 35–66, median 53; bone: 23–74, median 54). In the dentin group, there were slightly more males than females (65% vs 35%) whereas in the bone group, there were slightly more females (56% vs 44%). Baseline medical conditions were similar in the two groups with the highest frequency disorders being vascular (ca 20%), immune (ca 20%), metabolic (ca 20%), and psychiatric (ca 17%). Thirty-six subjects completed the study up to the time of implant placement (20 with dentin and 16 with bone). Three subjects from the dentin group discontinued, due to graft failure (*N* = 2) or to the investigator’s decision of anticipated non-compliance (*N* = 1). Two subjects from the bone group discontinued, one due to graft failure and the other due to consent withdrawal.

**Fig. 2:**
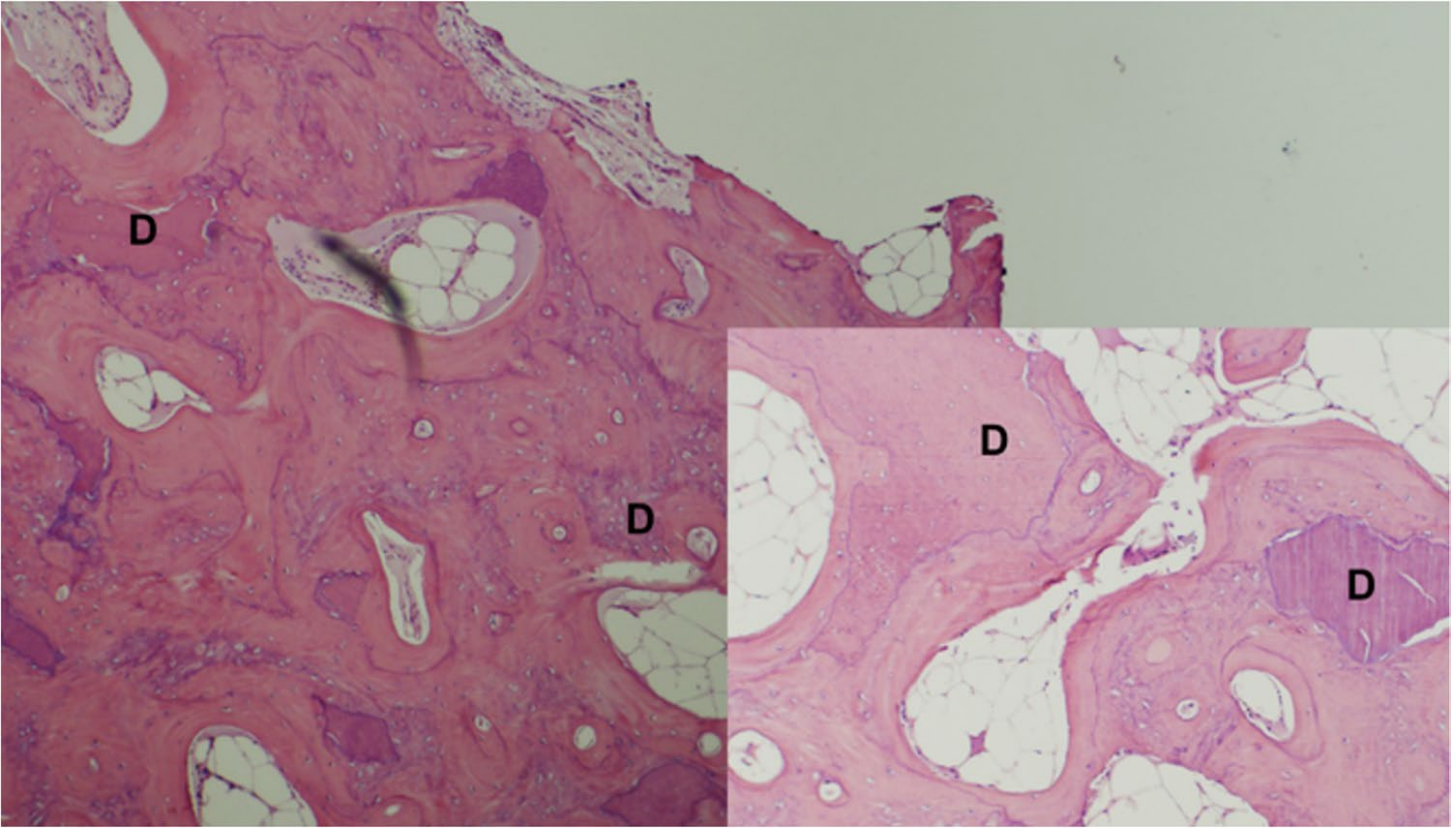
Histology of dentin graft site. Low magnification with higher magnification inset. Dentin particles are closely integrated with the bone. High magnification shows direct contact ankylosis-like contact. D, dentin

The mean amount of new bone formation (“woven bone”) in alveolar bone core biopsies from the dentin group was higher than for the bone group (60.75% vs 42.81% respectively, Table 3), and this difference was statistically significant (*p* = 0.0084). Furthermore, the majority (85%) of dentin group biopsies were rated as having good bone–graft integration compared to less than half (40%) of the bone group biopsies (Table 3, Fig. 2); this difference was statistically significant (*p* = 0.0066). These assessments were made by independent blinded assessors. The histology showed close contact of the dentin graft particles with abundant new host bone growth (Fig. 2) with interdigitation of the two in some areas. Ankylosis was also confirmed (Fig. 2).

**Table 3:** Comparative efficacy

Outcome [unit]	Ivory Dentin Graft™ (*N* = 20)	Gen-Os® (*N* = 16)
New bone formation [%, mean (SD)]	60.750** (18.229)	42.812 (17.413)
Alveolar strength [torque Ncm, mean (SD)]	34.750 (9.662)	34.062 (8.797)
Alveolar bone radiodensity [HU, mean (SD)]	981.500** (233.968)	727.688 (193.464)
Alveolar bone height change [mm, mean (SD)]	− 1.029 (2.213)	− 0.462 (1.897)
Alveolar bone width change [mm, mean (SD)]	− 0.430 (1.235)	− 0.331 (1.411)
Bone-graft integration	*N* = 20	*N* = 15
- Poor	5% (1)	26.67% (4)
- Intermediate	10% (2)	33.33% (5)
- Good	85% (17)	40% (6)
Implant placement success	95% (19/20)	81.25% (13/16)

**Statistically significantly different from Gen-Os at *p* < 0.05, Mann–Whitney test

**Fig. 3:**
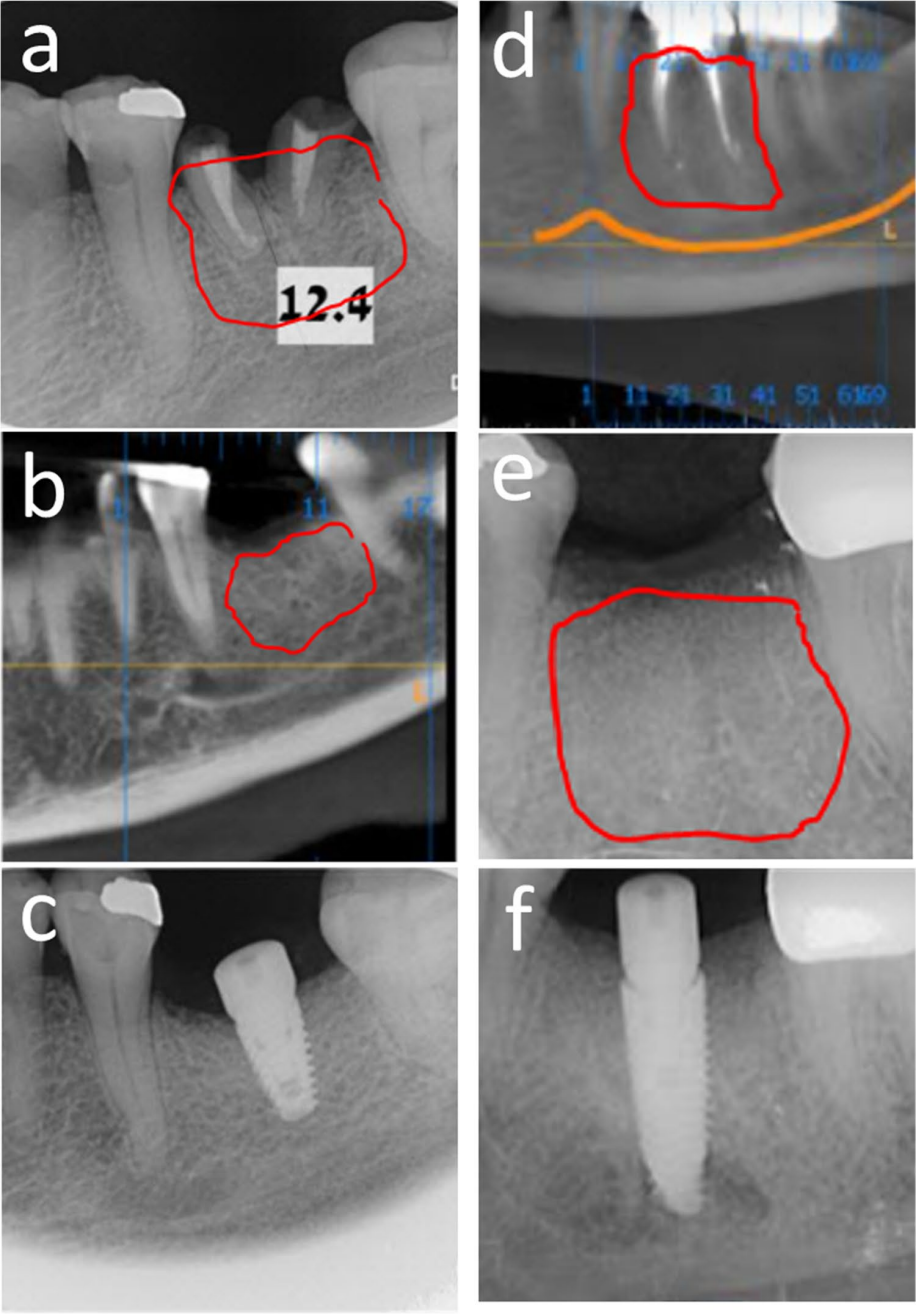
Example radiographs of graft sites. **a** Gen-Os® prior to extraction, **b** after grafting, and **c** after implant placement. **d** Ivory Dentin Graft™ prior to extraction, **e** after grafting, and **f** after implant placement

The mean radiodensity of the bone at the graft site was statistically significantly higher (*p* = 0.0011) for the dentin group (981.5 HU) compared to the bone group (727.7 HU) (Table 3). Example radiographs are shown in Fig. 3.

The mean torque force for implant placement at the graft site was similar for dentin (34.75 Ncm) and bone (34.06 Ncm) (Table 3).

The placement of a standard titanium implant at the dentin graft site was successful in 95% of subjects compared to 81.25% for the bone graft. There was a trend for a higher success rate with the dentin graft, but this difference was not statistically different as was to be expected as the study was not statistically powered for this endpoint.

No serious adverse events (SAEs) were recorded during the follow-up period. There were also no severe AEs, severe adverse reactions, or suspected unexpected serious adverse reactions (SUSARs). Overall, there were 28 adverse events in 16 subjects, with similar occurrence in both groups. Graft-associated events such as graft failure and graft complications occurred at a similar frequency in both groups, classified as mild for dentin and moderate for bone. There was also no difference in adverse events associated with local site reactions (local pain, swelling, tissue irritation, and erythema) most of which were mild and transient.

The physician assessment of the usability of dentin versus bone, as measured by a 10-point scale, in terms of either ease of grafting (8.78 ± 0.79 vs. 8.27 ± 1.44, mean and SD) or ease of dental implant placement (8.05 ± 1.43 vs. 8.75 ± 1.12, mean and SD) revealed no statistically significant differences (*p* = 0.2355 and 0.1118, respectively, Mann–Whitney test). This suggests that the physician experience with Ivory Dentin Graft is not inferior than the experience with a similar device in the market, Gen-Os®.

## Discussion

Following tooth extraction, the alveolar ridge bone starts to resorb resulting in reductions of both the height and width of the bone walls surrounding the socket, which can make the placement of a stable implant difficult [38, 39]. These changes occur most rapidly over the first 3 months after extraction but continue after this period. Bone graft materials have therefore been used to preserve the ridge dimensions and form a stable bony substrate for implant insertion [7, 21, 38, 40].

Ivory Dentin Graft™ is a novel bone graft material that potentially offers advantages over existing materials [41]. It is a particulate material with a similar particle size distribution (300–900 µm) to other established graft materials such as Bio-Oss® or Gen-Os®. The particles are, however, produced from porcine tooth dentin rather than bone. Dentin is a relatively compact material that offers good structural stability and is more slowly resorbed than bone. Dentin, in addition, can form close contact with regenerating bone in a process of ankylosis such that it rapidly establishes a mechanically stable scaffold with the ingrowing bone thus maintaining the graft site and providing a good substrate for dental implant placement [18, 19]. The physico-mechanical properties of dentin, such as compressive and tensile strength and elasticity (Young’s modulus) [42–50], are either similar to that of cortical bone or even superior (Table 4) and thus can form a stable scaffold with the regenerating bone. Dentin is characterized by a regular arrangement of tubular pores with dimensions of 1–2 µm which is an ideal size for interaction with host tissue while bone generally has pores that are larger or smaller than this range. Autogenous tooth dentin has been successfully used as a bone graft material for dental procedures [9–17, 51, 52] confirming that dentin-based materials are suitable for grafting but having the disadvantage that there is often not enough material for the required procedure. Ivory Dentin Graft™ is processed at relatively low temperatures compared to materials such as Bio-Oss® and thus retains the protein content of the dentin (ca. 34%), which is largely collagen but also contains growth factors [9, 17, 22, 23]. The protein content thus promotes the ingrowth of host tissue. Based on these dual processes of rapid host tissue ingrowth and the establishment of a mechanically stable interaction between host bone and the material, it was expected that Ivory Dentin Graft™ could establish a stable implant site following tooth extraction relatively early after grafting.

**Table 4:** Physico-mechanical properties of dentin compared to bone and other tooth structures

Anatomical structures	Compressive strength (MPa)	Tensile strength (MPa)	Elasticity (Young’s modulus, GPa)
Dentin	295	52 /103	18.6
Cortical bone	88–165	89–114	13.7
Spongeous bone	N/A	N/A	1.37
Bone	167	123	N/A
Enamel	321/384	16.7	EX = 80 EY = EZ = 20
Pulp	2.94	2.94	0.02

In the area of dental bone graft materials, it has been criticized that there is a lack of high-quality clinical trials to support the use of particular materials [21, 38]. The current clinical study examining the performance and safety of Ivory Dentin Graft™ in comparison to a commercially used graft material was thus performed to appropriately examine its properties. Care was therefore taken to perform a randomized controlled clinical trial that conforms to CONSORT guidelines.

The reference product Gen-Os® (Tecnoss) was considered appropriate because it is a porcine bone-derived graft material with retained organic matrix that has been shown to be effective in a number of clinical studies [38, 53] and is as good as Bio Oss®.

In keeping with the expected early graft stability, the histological assessment of the graft site and implantation were performed 4 months after grafting, which is comparatively early. The study was statistically powered to examine non-inferiority to the reference material in terms of the histological condition of the graft site in terms of the area of the host woven bone and also the extent of direct contacts between the host bone and the graft particles. Additional relevant parameters were also examined. The subjects included in the study were typical for those seen in clinical practice with the main exclusion criteria consisting of conditions that would be contraindications for bone grafting. A consistent grafting procedure was used for all study subjects which included the use of a collagen membrane (Janson® fleece) to hold the graft material in place and exclude soft tissue infiltration. A semi-blinded procedure was used where the person performing the procedure was unblinded in order to use the material appropriately, but the clinical and histological assessors were unaware of the material used.

The clinical study comparing Ivory Dentin Graft™ with Osteobiol Gen-Os® (Tecnoss) fulfilled the primary outcome of demonstrating that Ivory Dentin Graft™ is not inferior to the comparator in terms of the quantity and quality of regenerated bone at the graft site at 4 months after grafting. In fact, the mean percentage of new bone formation for Ivory Dentin Graft™ of 60.75% was considerably higher than that of the comparator which had just 42.81% of new bone. Comparison with other studies [24, 28, 38] shows that the new bone growth for the comparator is in the range for other materials seen at 3–6 months and even superior to that seen for some materials. This therefore suggests that the dentin-derived material with retained protein encourages superior new bone growth compared to other materials, including various synthetic materials, xenogeneic materials, and even autologous bone marrow. Furthermore, a qualitative assessment of the degree of close contact between the graft particles and the new bone clearly showed that a higher proportion of subjects receiving Ivory Dentin Graft™ (17/20) had good host bone-graft integration compared to the comparator (6/15). The histological data therefore confirms the presumption that Ivory Dentin Graft™ establishes a stable implant site already at 4 months after grafting.

Consistent with the excellent new bone growth for Ivory Dentin Graft™, the graft sites had a significantly higher radiodensity than the comparator (981.5 HU vs. 727.7 HU). This difference did not, however, translate into a difference in mean insertion torque which was similar for Ivory Dentin Graft™ and the comparator (34.75 Ncm vs. 34.06 Ncm). The higher radiodensity probably reflects the higher density of the dentin-derived graft material. It has been shown that in this intermediate range of bone densities and insertion torques, there is no statistical correlation between the parameters [54], and so this is not unexpected. Insertion torques above ca. 30 Ncm are consistent with stable implants that have a good outcome, and so both products are in an acceptable range of insertion torque [55]. The higher proportion of new bone in the Ivory Dentin Graft™ sites may, however, potentially lead to more rapid implant integration.

Sufficient ridge width and height have been considered one of the key requirements to ensure the longevity and function of implant-supported prosthesis [56]. Since the bone resorption process is initiated immediately after extraction, leading to an average 40–60% decrease in the horizontal and vertical dimensions of the alveolar ridge, during the first 2 years [57], it is imperative to preserve the alveolar ridges to provide adequate bone volume for successful implant placement. The analysis of the changes from baseline in alveolar ridge dimensions (height and width) at 4 months post grafting showed that there are no significant differences between the investigational and the comparator group in vertical and horizontal bone resorption during this period (height change: *p* = 0.5881 and width change *p* = 0.61).

These data are in line with data presented in other clinical investigations assessing dimensional alterations of the alveolar ridge at 4 months following bone grafting. The mean vertical loss at 4 months following tooth extraction and grafting with porcine (Gen-Os® and Deproteinized Porcine Bone Mineral (DPBM)) and bovine xenografts (Bio-Oss® and Deproteinized Bovine Bone Mineral (DBBM)) ranged between 0.5 and 1.45 mm [53, 54], and the horizontal loss at 4–8 months post-grafting with DBBM or cortico-cancellous porcine (Gen-Os®) was 1.07–1.6 mm [55, 56], both well in line with the mean height and width measured for the Ivory Dentin Graft™ group in this study (mean height change − 1.029 mm and mean width change − 0.43 mm).

Although the bone remodeling process continues for months after the grafting procedure, these short-term efficacy data indicate that Ivory Dentin Graft™ is not inferior to Gen-Os® in providing adequate bone volume to support implant placement. Furthermore, as the dentin particles are resorbed slowly by sterile external replacement resorption, the stability is expected to be maintained throughout the resorption process.

Consistent with the excellent data for Ivory Dentin Graft™ concerning the graft site, the implant placement success was 95% compared to 81.25% for the comparator. This indicates that most implants did not require any additional intervention during implant placement or delaying of implant placement due to graft failure. Physician assessment of the usability of the products using a 10-point satisfaction scale indicated that the materials are similarly easy to use.

Safety aspects were actively monitored throughout the study. There were no serious AEs, and also no severe adverse events, severe adverse reactions, or suspected unexpected serious adverse reactions for either product. Importantly, there was no difference in the incidences of probably and possibly product-related AEs.

Most AEs were of the type already described for such oral surgical procedures, were mild to moderate, and resolved without consequences. Overall, the safety of Ivory Dentin Graft™ was excellent, with an AE and tolerability profile similar to that of the comparator and as would be expected for this type of therapy.

This clinical investigation therefore confirmed that Ivory Dentin Graft™ is efficacious and safe in providing an adequate site for implant placement at 4 months after tooth extraction and is non-inferior to the commercially used product Gen-Os® in terms of new bone growth and graft-host integration at the grafting site. Ivory Dentin Graft™ provided adequate alveolar ridge preservation which allowed stable implant placement and at the graft site had a higher proportion of new bone growth and integration of the graft with the new bone than Gen-Os®. These properties are consistent with the unique aspect of Ivory Dentin Graft™ being based on tooth dentin with a retained protein component. The high proportion of new bone growth at the time of implantation combined with the structural strength of the dentin and intimate bone-graft contact provides for rapid and stable integration of the implant that, however, needs confirmation at later time points. This early follow-up clinical data thus suggests that Ivory Dentin Graft™ is suitable to extend the use of dentin-based bone graft material beyond that of autologous procedures.

### Study limitations

This study was designed to provide a sensitive comparison of the properties of a novel dentin-derived graft material with an already established bone-derived material under standardized conditions. The enrolment criteria thus excluded patients with comorbidities that may have independently influenced the outcome. These criteria are, however, generally contraindications for implant placement. Only mandibular tooth extraction sites were grafted, and therefore, the outcomes need to be adjusted for other graft locations and situations. The comparator material has, however, been extensively tested in other procedures including sinus lifting, and the principles of host bone ingrowth and formation of a stable site are expected to be similarly transferable for the dentin material. For the histological assessment, only one timepoint could be assessed which is at the time of implant placement at 4 months. Good graft site stability was demonstrated. Subsequent long-term clinical follow-up is required to confirm that this translates into long-term implant stability.
